# Collapsin response mediator protein 5-associated optic neuropathy: clinical characteristics, radiologic clues, and outcomes

**DOI:** 10.3389/fneur.2023.1163615

**Published:** 2023-06-28

**Authors:** Rong Yan, Yu Mao, Huiyang Zeng, Qian Liu, Hanqiu Jiang, Jingting Peng, Qingling Yang, Shilei Cui, Lei Liu, Yanjun Guo, Jiawei Wang

**Affiliations:** ^1^Department of Neurology, Beijing Tongren Hospital, Capital Medical University, Beijing, China; ^2^Department of Ophthalmology, Beijing Tongren Hospital, Capital Medical University, Beijing, China; ^3^Institute of Ophthalmology, Beijing Tongren Hospital, Capital Medical University, Beijing, China

**Keywords:** anti-CRMP5 autoantibody, magnetic resonance imaging, optic neuritis, paraneoplastic neurologic syndromes, autoimmune optic neuropathy

## Abstract

**Objective:**

Collapsin response mediator protein 5-associated optic neuropathy (CRMP5-ON) is a rare entity of autoimmune optic neuropathy. This study aimed to review the neuro-ophthalmic findings and outcomes in a series of patients with CRMP5-ON to further characterize its clinical phenotype, radiologic clues, and outcomes.

**Methods:**

This was a retrospective case series and a single-center medical chart review of all patients with CRPM5-seropositive ON at the Department of Neurology, Beijing Tongren Hospital, from December 1, 2020, to March 31, 2023. The main outcome measures were neuro-ophthalmic manifestations, radiologic characteristics, and clinical outcomes of CRMP5-ON; coexisting neural autoantibody, paraneoplastic associations, and the impact of immunosuppressant therapy.

**Results:**

Five patients were identified. Four (80%) were female, and the average age at onset was 59.4 years (range 53–69 years), with an average follow-up of 15.3 months (range 1.4–28.7 months). The average best-corrected visual acuity (BCVA) at nadir was 20/120 (range 20/20 to count fingers). Seven of ten affected eyes (70%) showed diffuse defects of the central field. Painless bilateral involvement and optic disk edema occurred in 100% of patients, combined with vitritis, uveitis, or retinitis in four (80%). Four patients (80%) had MRI abnormalities along the optic nerve (one patient with optic nerve enhancement and three patients had optic nerve sheath enhancement or peribulbar fat enhancement). Three patients (60%) had optic neuropathy with other neurologic symptoms. Four patients (80%) had confirmed cancer (two were small-cell lung carcinoma, one was papillary thyroid carcinoma and another was thymoma and invasive pulmonary adenocarcinoma). All cancers were identified after the presentation of the optic neuropathy. The intervention included IVIG, IVMP, surgery and chemotherapy. The average BCVA at the last follow-up was 20/50 (range 20/20 to count fingers). Three patients had surgery during the initial hospitalization, and were stable during the follow-up. Among two patients who received IVMP, both had improvement after treatment, although one patient had worsening non-ocular neurologic symptoms during the steroid taper.

**Conclusion:**

CRMP5-ON presented with optic disc edema, often bilateral involved and combined with vitreitis, retinitis, or uveitis. CRMP5-ON can present with MRI optic nerve or perineural optic nerve enhancement, especially in the optic nerve sheath. CRMP5-ON is closely related to paraneoplastic neurologic syndrome. Cancer screening and intervention are crucial to prognosis.

## 1. Introduction

Currently, optic neuritis has entered the era of biomarkers (1). Patients with optic neuropathy have three clinically validated and specific autoantibodies, binding to aquaporin 4, myelin oligodendrocyte glycoprotein (MOG), and collapsin response mediator protein 5 (CRMP5), which are considered helpful in diagnosing and classifying optic neuritis (2). Recently, Petzold et al. provided a consensus view on the diagnosis and classification of optic neuritis (2). They proposed a new classification of autoimmune optic neuritis, including MS-associated optic neuritis (MS-ON), AQP4 antibody–associated optic neuritis (AQP4-ON), MOG antibody–associated optic neuritis (MOG-ON), and CRMP5 antibody–associated optic neuritis (CRMP5-ON). Compared with typical optic neuritis (usually MS-ON), atypical optic neuritis often presents as bilateral simultaneous or rapidly sequential involvement，severe optic disk swelling, painless and severe visual loss. Some patients have a family or neoplastic history，needing prolonged immunosuppressive regimens (3).

The collapsin response mediator protein (CRMP) family comprises five cytosolic phosphoproteins strongly expressed in the developing nervous system. CRMP5 is mainly expressed in oligodendrocytes, olfactory bulbs, olfactory epithelium, retina, hippocampal dentate gyrus, peripheral nerve axons, and sensory neurons (4). In 1996, Honnorat et al. found that the CV2 antibody was specific for oligodendrocytes and considered that the target antigen was CRMP3 (66 kDa); they were the first to clarify the relationship between CV2 and paraneoplastic neurologic syndrome (PNS) (5). In 2001, Mayo Clinic reported 116 CRMP5-seropositive patients (7% with optic neuropathy). The CV2 antibody was found to be actually specific for CRMP5 (62 kDa) and considered a biomarker of lung cancer and thymoma-related paraneoplastic syndrome (6). CRMP5-ON, as a kind of paraneoplastic optic neuropathy (PON), is associated with cancer and PNS, which is rarer than AQP4-ON and MOG-ON clinically. As previously reported, CRMP5-ON typically presents with painless, bilateral, and subacute progressive vision loss, almost always combined with vitritis, retinitis, or both, but it rarely causes enhancement of the optic nerve on MRI (7, 8). Here we reported a cohort of patients with CRMP5-ON in China with different MRI features of the optic nerve to further characterize this disease.

## 2. Methods

This study was approved by the ethics committee of Beijing Tongren Hospital, and informed consent was exempted because this study was a retrospective case series. We reviewed all the clinical records of patients with optic neuropathy identified as seropositive for the CRMP5 antibody from the database of the Beijing Tongren Hospital (December 1, 2020, to August 31, 2022). These patients were diagnosed with CRMP5 antibody–associated optic neuropathy (CRMP5-ON) according to the 2022 diagnosis and classification of optic neuritis (2), combined with the clinical manifestations and laboratory examination at baseline. The diagnostic criteria of PNS were followed by the updated diagnostic criteria for PNS and PNS-CARE score (9). The findings of demographic, clinical, pathologic, and neuro-ophthalmology examinations were tabulated, which included best-corrected visual acuity (BCVA) at presentation and final follow-up (with Snellen conversion to the logarithm of the minimum angle of resolution for analysis), poor VAs ≤20/400 (such as “count fingers” or “hand movements”) is defined as 1.3 LogMAR for analysis. Humphrey visual field，optical coherence tomography (OCT), fluorescein fundus angiography (FFA), and magnetic resonance imaging (MRI) of the brain and optic nerve. The MRI and positron emission tomography (PET-CT) of the spinal cord were partially obtained.

The blood tests included antinuclear antibody, anti-double-stranded DNA, anti-Sm, Sjogren syndrome A or Sjogren syndrome B, anticardiolipin (ACL and β2-GPI), antineutrophil cytoplasmic antibodies, rheumatoid factor, and human leucocyte antigen B27 phenotype. The cerebrospinal fluid (CSF) samples were used for the routine testing of white cell counts, total protein levels, concentrations of immunoglobulin G (IgG), and cytopathology. The serum AQP4 antibody and MOG antibody were detected using the cell-based assay. The PNS antibody examinations in serum, including ANNA-1/anti-Hu, PCA-1/Yo, ANNA-2/Ri, Ma2, CRMP5, amphiphysin, recoverin, and SOX1 antibodies, were performed using Western blot assay. Autoimmune encephalitis antibodies were detected using indirect immunofluorescence testing, including anti-N-methyl-D-aspartate receptor, leucine-rich glioma inactivating 1 protein, contact protein–associated protein (Caspr2), α-amino-3-hydroxy-5-methyl-4-isoxalpropionic acid receptor, and γ-aminobutyric acid type B receptor antibody. The serum of two patients with retinitis were sent to the Beijing Institute of Ophthalmology, Beijing Ophthalmology & Visual Sciences Key Laboratory, for the detection of anti-retinal antibodies (α-enolase, CRMP5, recoverin and carbonic anhydraseII etc.) using Western blot assay before treatment.

## 3. Results

### 3.1. Clinical features and oncological characteristics

A total of five patients (four female and one male) diagnosed with CRMP5-ON were identified. The age ranged from 53 to 69 years (average 59.4 years). Table 1 summarizes the visual manifestations, neurological accompaniments, imaging and spinal fluid abnormalities, coexisting neuronal autoantibodies, type of cancer, and clinical course. Patient 1 had a history of colon polypectomy, patient 2 was a smoker with a history of Brucella infection, patient 4 had a history of deep vein thrombosis (DVT), and patient 5 had a history of Hepatitis C and atopic dermatitis. The duration of vision loss at nadir was 7–10 days in three patients and 2 months in two patients. Active cancer was confirmed in four patients (80%) histologically: two had small-cell lung carcinoma, one had papillary thyroid carcinoma, and another was thymoma and invasive pulmonary adenocarcinoma. Patient 4 who lacked the confirmation of cancer showed elevated serum levels of squamous cell carcinoma–associated antigen and had a history of DVT. All cancers were diagnosed after the presentation of the optic neuropathy, and the average time from optic neuropathy to cancer diagnosis was 5.8 months (ranging 1–12 months).

**Table 1:** Baseline clinical characteristics of five patients with CRMP5-ON.

Case	Sex	Age^a^	BCVA at nadir	Bilateral interval time	Duration of vision loss at nadir	Visual field at nadir	Ophthalmic findings
1	F	59	OD 20/20 OS 20/63	14 days	10 days	OD: enlarged blind spot OS: quadrant defect	Optic disc edema, vitritis, uveitis
2	M	69	OD 20/400 OS 20/32	10 months	OD:NA OS:2 months	OU: diffuse central scotoma	Optic disc edema, vitritis, retinitis, CNV
3	F	53	OD 20/100 OS FC	Simultaneous	10 days	OU: diffuse central scotoma	Optic disc edema, vitritis
4	F	55	OD 20/200 OS 20/63	3 days	7 days	OD: diffuse central scotoma OS: peripheral defect	Optic disc edema
5	F	61	OD 20/200 OS FC	Simultaneous	2 months	OU: diffuse central scotoma	Optic disc edema, vitritis, retinitis

Case	Coexisting neural autoantibodies	Cancer	Time from visual symptoms to cancer diagnosis	Past history	Neurologic manifestations
1	ANNA1/anti-Hu	SCLC，with lymph node metastasis	1 months	Colon polypectomy	Painful polyradiculoneuropathy
2	ANNA1/anti-Hu，α-enolase	SCLC	OD:1 year OS:2 months	Smoke; brucella infection	None
3	SOX1	Papillary thyroid carcinoma， with lymph node metastasis	4 months	No	Painful polyradiculoneuropathy
4	Negative	Not found yet; Serum: SCC 2.1 ng/ml.^b^	None	DVT	Area postrema syndrome,^c^ myelopathy
5	α-enolase, CA II	Thymoma; Invasive pulmonary adenocarcinoma	10 months	Hepatitis C, atopic dermatitis	None

Case	Duration of visual loss to MRI	Optic nerve MRI	Brain and spinal cord MRI	OCT	CSF	PET-CT
1	20 days	T2 lesion on the left and optic chiasma, bulbar fascia enhancement	Brain: nonspecific, Spinal cord: NA	OS: peripapillary detachment	Pressure:150 mm H_2_O，WBC: 24 × 10^6^/L, protein: 67 mg/L, IgG synthesis rate: 45.7 mg/d Cytology: normal.	Left hilar hypermetabolic mass
2	1 year	Bilateral optic nerve thinning	Brain: nonspecific, Spinal cord: NA	OD: CNV, OS: epimacular membranes OU: peripapillary detachment	Normal	NA
3	20 days	Bilateral optic nerve sheath and optic disk enhancement	Brain: nonspecific, Spinal cord: none	OU: RD of posterior pole area	Pressure: 110 mm H_2_O，WBC: 36 × 10^6^/L, protein: 43.9 mg/L, IgG synthesis rate: 11.8 mg/d. Cytology: normal.	NA
4	44 days	Bilateral optic nerve sheath enhancement	Brain: nonspecific, Spinal cord: C4–6 T2 lesions	OD: pRNFL thickening OS: pRNFL normal	Pressure: 120 mm H_2_O, WBC: 4 × 10^6^/L, protein: 41.6 mg/L, IgG synthesis rate:1.34 mg/d. Cytology: normal.	NA
5	9 months	Left optic nerve enhancement	Brain: Left cerebellar meningioma, Spinal cord: NA	OU: atrophy of macula, loss of the inner segment/outer segment (IS/OS) junction	NA	NA

F, female; M, male; OD, right eye; OS, left eye; OU, both eyes; BCVA, best-corrected visual acuity; FC, finger counting; SCLC, small-cell carcinoma of the lung; CNV, choroidal neovascularization; RD, retinal detachment; RPE, retinal pigment epithelium; pRNFL, peripapillary retinal nerve fiber layer, CA II: carbonic anhydraseII.

a Age at onset (year).

b Squamous cell carcinoma–associated antigen (SCC), reference:0–1.5 ng/m.

c Area postrema syndrome (APS): intractable nausea vomiting and hiccups, self-relieved after 1 month.

### 3.2. Ophthalmic findings

**Figure 1:**
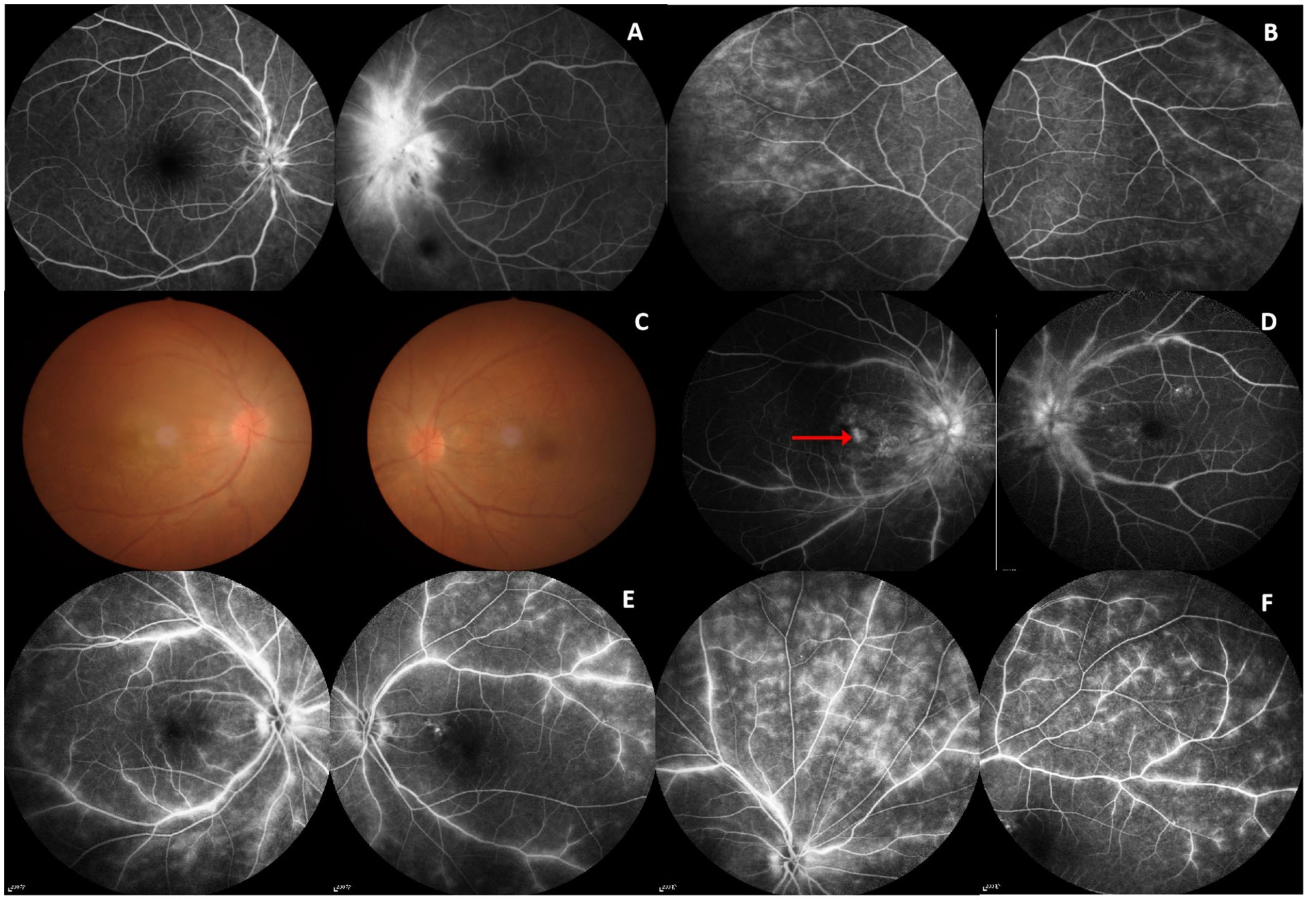
FFA and fundus image. Patient 1 (F, 59 years): **(A)** FFA: mild disk edema in the right eye and disk edema in the left eye with small hemorrhage. **(B)** FFA: Bilateral retinal telangiectasia and fluorescence leakage. Patient 2 (M, 69 years): **(C)** Fundus image: Bilateral disk edema and vitreous opacity. **(D)** FFA: Bilateral disk edema, retinal vascular staining, and leakage; late frame of the right eye showing hyperfluorescence in the macular area caused by leakage from choroidal neovascularization (CNV; red arrowed). Patient 5 (F,61 years): **(E,F**) FFA: Bilateral hyperfluorescence and leakage of the optic disc and retinal.

All patients were bilateral involvement without pain: two were simultaneous and three were sequential. Seven of 10 affected eyes (70%) showed a diffuse defect of the central field, and the remaining had peripheral field defect, quadrant field defect, and blind spot enlargement, respectively. The average BCVA at nadir was 20/120 (range from 20/20 to count fingers). All patients had optic disk edema, four of five (80%) with associated vitritis, retinitis, or uveitis, only one (patient 4) had isolated optic disc edema without other ocular findings (Table 1). Patient 1 had bilateral vitritis and uveitis (Figures 1A,B). Patient 2 had bilateral vitritis, retinitis and right eye choroidal neovascularization (CNV; Figures 1C,D). Patient 5 had bilateral vitritis and retinitis (Figures 1E,F).

### 3.3. Neurological manifestations

Three patients (60%) had other neurological symptoms or signs during their illness (Table 1). One had painful polyradiculoneuropathy 2 months before ON, one had painful polyradiculoneuropathy 6 months before ON and then myelopathy and cerebellar ataxia 4 months after ON, and one had area postrema syndrome 3 months before ON and myelopathy 1 month before ON.

### 3.4. Imaging findings

**Figure 2:**
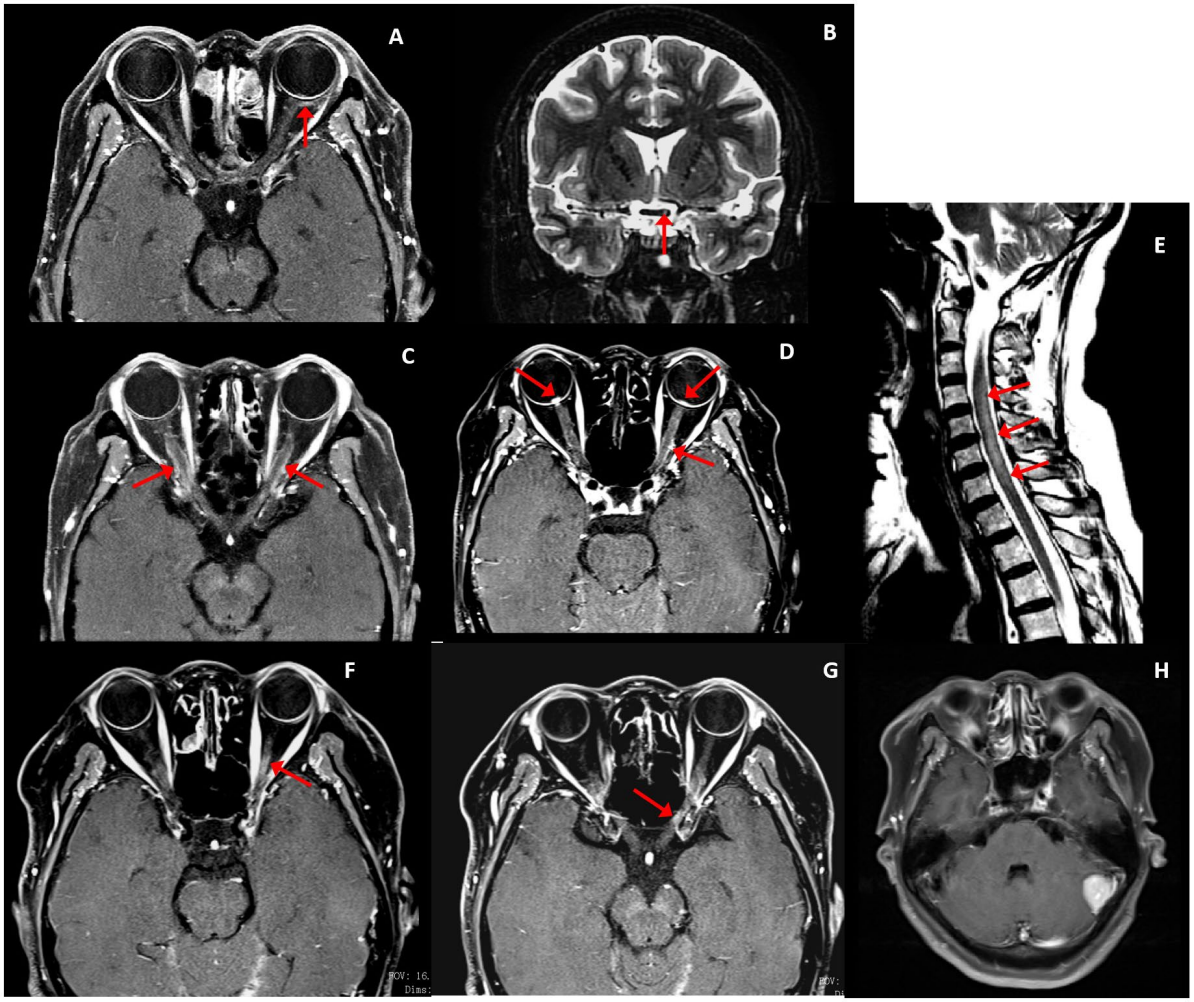
Optic nerve and spinal cord MRI (red arrowed). Patient 1 (F, 59 years): **(A)** Post-gadolinium axial T1 MRI demonstrated left bulbar fascia mild enhancement. **(B)** T2 lesion on the left side of the optic chiasma. Patient 4 (F, 55 years): **(C)** Post-gadolinium axial T1 MRI demonstrated bilateral optic nerve sheath mild enhancement. Patient 3 (F,53 years): **(D)** Bilateral optic nerve sheath mild enhancement and optic disk enhancement. **(E)** C2–7 T2 lesions. Patient 5 (F,61 years): **(F,G)** Left optic nerve enhancement. **(H)** Left cerebellar meningioma.

Four of five (80%) patients had optic nerve or perineural optic nerve enhancement. Only one (patient 2) with MRI performed 1 year after initial visual loss showed bilateral optic nerve atrophy and thinning without T2 lesions or enhancement. The others had MRI within 9 months since visual loss presented with enhancement (patient 1 had left bulbar fascia (retro-ocular orbital fat) enhancement and T2 lesion on the left side of optic chiasma without corresponding enhancement; patient 3 and 4 had bilateral longitudinally optic nerve sheath enhancement; patient 5 had left optic nerve enhancement). Two patients had myelopathy. Spinal cord MRI showed T2 lesions of the involved spinal cord with T1 mild enhancement. Patient 5 was found a meningioma in the left cerebellar (Figures 2A–H). Of the five patients, two completed the PET-CT examination: patient 1 showed a hypermetabolic mass in the left hilum of the lung; patient 3 showed a hypermetabolic nodule in the left lobe of the thyroid and a local metabolic increase in the left cerebellar hemisphere and cervical spinal cord during the follow up.

### 3.5. Laboratory findings

All of the serum samples were negative for the AQP4 and MOG antibodies, and no one had abnormal serum autoimmune antibodies. The coexisting neural autoantibodies included antineuronal nuclear-1 (ANNA-1/anti-Hu; 2, 40%), SOX1 (1, 20%), α-enolase (1, 20%), and α-enolase with carbonic anhydraseII (1, 20%). The cerebrospinal fluid (CSF) demonstrated an elevation of white blood cell (WBC) count in two patients (50%) and an elevation of protein and intrathecal IgG synthesis in three patients (75%). The CSF pressure and cytology were initially normal. During the follow-up after an outpatient visit, one patient developed myelopathy and cerebellar ataxia. The CSF pressure and the synthesis rates of protein and intrathecal IgG were higher than earlier, and a few degenerative cells were found on cytological examination.

### 3.6. Clinical outcomes

The average follow-up time was 15.3 months, ranging from 1.4 to 28.7 months. The average BCVA at the last follow-up was 20/50 (range 20/20 to count fingers). The visual acuity of patient 1 did not improve after IVIG, then the patient had surgery and chemotherapy. At 28.7 months of follow-up, the patient’s visual acuity was stable, cancer did not recur, and no other neurological symptoms appeared. Patient 2 only had surgery and chemotherapy, he was stable after 8.5 months follow-up. Only two patients (patient 3 and 4) received IVMP, their visual acuity improved after the treatment. But patient 3 developed myelopathy and cerebellar ataxia during the steroid taper. These symptoms were getting stable after she had surgery of thyroid. Patient 4 was followed up to 27.7 months, the dose of oral methylprednisolone was reduced to 2 mg/day. Her symptoms were stable, and no cancer was found yet. Patient 5 only had surgery, and her visual acuity was stable so far (Table 2).

**Table 2:** Intervention measures and follow-up data.

Case	ON intervention	Cancer intervention	Follow-up
1	IVIG	surgery and chemo	At 28.7 months: stable.
2	None	surgery and chemo	At 8.5 months: stable.
3	IMVP IVIG	None	At 4 months: BCVA OD1.0/OS0.7，myelopathy and cerebellar ataxia occurred. Spinal cord MRI: C2-7 T2 lesions and mild enhancement. PET-CT: Thyroid hypermetabolic mass, left cerebellar hemisphere and cervical spinal cord metabolism increased. CSF: pressure 235mmH_2_O, WBC 36 × 10^6^/L, protein:82 mg/L, IgG synthesis rate:29.1 mg/d, cytology: degenerated cells. Papillary thyroid carcinoma was confirmed by biopsy. Surgery after that, cancer pathology: papillary thyroid carcinoma. At 10.2 months: stable.
4	IMVP	None	At 27.7 months: BCVA OU1.0, no cancer was found.
5	None	surgery	At 1.4 months: stable.

IVIG, Intravenous immunoglobulin, 0.4 g per kg body weight for five consecutive days. IMVP: intravenous methylprednisolone, 500–1,000 mg/d for three to five consecutive days and continuous dose of oral prednisolone (1 mg/kg) gradually decreased. Chemotherapy: carboplatin and etoposide.

## 4. Discussion

In 2003, Cross et al. reported 13 patients with CRMP5-seropositive optic neuropathy with retinitis and/or vitritis (7). The orbital MRI was infrequently performed in these earlier studies, therefore the actual incidence of optic nerve enhancement was not known. In 2019, Cohen et al. reported 14 patients with CRMP5-IgG-positive optic neuropathy with optic disk edema, often associated with uveitis and/or retinitis, but without optic nerve signal enhancement (8). Within our CRPM5-ON cohort, all patients presented with painless, bilateral involvement and optic disk edema. Only 1 of our patients with optic disc edema did not have accompanying vitritis or retinitis. Most of them had vitritis, retinitis, or uveitis, which were similar to the previously reported findings; so did the demographic characteristics, coexisting neural autoantibodies, and cancer type (7, 8). However, unlike previous studies (7, 8), most of our patients had optic nerve or perineural optic nerve enhancement (Table 3). Enhancement of optic nerves has been reported in CRMP5 optic neuropathy in some case reports (10, 11). Pathologically, perivascular lymphocytic infiltration in the optic nerve is found along with axonal demyelination, and immunohistochemical studies revealed CD8+ T-cells infiltrating the optic nerve (7). Therefore, CRMP5-ON can present with MRI optic nerve or perineural optic nerve enhancement, especially in the optic nerve sheath. Unlike the typical optic nerve enhancement seen in other causes of optic neuritis (MS, MOGAD, and NMOSD), the enhancement of CRMP5-ON is milder. We assumed that early investigation and the advance orbital MRI techniques and protocols used (2) might increase sensitivity of MRI to detect active inflammation within or around the optic nerve.

**Table 3:** Published cohort of CRMP5-ON versus our cohort.

Year	First author	Case	female (%)	Age	Subacute (%)	VA at nadir	Bilateral involved (%)	Optic disk edema (%)
2003 (7)	Cross	13	53.8	67.2	61.5	NA	46.2	69.2
2019 (8)	Cohen	14	71.4	65.4	78.6	20/60	85.7	85.7
2023	Yan	5	80	59.4	40	20/120	100	100

Ocular findings	Other manifestations	Coexisting autoantibodies	MRI enhancement	Cancer
Vitritis, uveitis, and retinitis	Peripheral neuropathy, cerebellar ataxia，cognitive decline，myelopathy，autonomic dysfunction，and DVT	VGCC, ANNA-1, amphiphysin, and GAD65	Optic nerve: No Spinal cord: Yes	SCLC, renal carcinoma, and papillary thyroid carcinoma
Vitritis, retinitis, and iritis	Peripheral neuropathy, cerebellar ataxia，cognitive decline，myelopathy，and autonomic dysfunction	PCA2, GAD65, VGCC, amphiphysin, ANNA-1, and ANNA-3	Optic nerve: No Spinal cord: NA	SCLC, Mixed SC/NSCLC
Vitritis, uveitis, retinitis, and CNV	Peripheral neuropathy, cerebellar ataxia，myelopathy, area postrema syndrome, and DVT	ANNA1,SOX1,α-enolase, CA II	Optic nerve: Yes Spinal cord: Yes	SCLC, papillary thyroid carcinoma, thymoma, pulmonary adenocarcinoma

We detected serum autoantibodies against α-enolase and carbonic anhydrase II in two of our patients, but not recoverin. The α-enolase and carbonic anhydrase II antibody are anti-retinal antibody (ARA), which in combination with clinical manifestations have been proposed to have important diagnostic value for confirmation of autoimmune retinopathy (12). However, a recent study showed these ARA’s can be present in up to 2/3 of normal individuals, and therefore are fairly nonspecific (13). Most likely, the retinopathy in these cases was related to the CRMP5 antibody, which has been shown to cause retinitis in addition to optic neuropathy and vitritis. Patient 5 did not test electroretinography, but her OCT showed loss of the inner segment/outer segment (IS/OS) junction, and FFA revealed hyperfluorescence and leakage of the optic disc and retinal. Combined with clinical manifestations we diagnosed the patient CRPM5-ON with cancer-associated retinopathy (CAR).

In 2021，the PNS-Care panel updated the diagnostic criteria for PNS and developed a scoring system (PNS-Care Score); paraneoplastic retinopathy and optic neuropathy are not included within the spectrum of PNS (9). PNS of CRMP5 had high clinical heterogeneity. Painful axonal asymmetric polyradiculoneuropathy was established as the major CRMP5 autoimmune neuropathy presentation (65%). The other neurologic associations were cerebellar ataxia (21%), myelopathy (19%), optic neuropathy, and/or retinitis (11%) (14). Within our cohort, 60% of patients had other symptoms of nervous system involvement besides the optic nerve: patient 1 had peripheral neuropathy before ON, complicated with SCLC, PNS-Care scored 10; patient 3 had peripheral neuropathy before ON and myelopathy and cerebellar ataxia after ON, complicated with papillary thyroid carcinoma, PNS-Care scored 10; and patient 4 with a history of DVT had area postrema syndrome and myelopathy before ON, no cancer was found with a follow-up of less than 2 years, but the serum level of squamous cell carcinoma–associated antigen was elevated, PNS-Care scored 7. The first two cases met the diagnostic criteria of definite PNS, and the third case was probable PNS. Therefore, CRMP5-ON can be regarded as a clinical manifestation of PNS, and patients with isolated CRMP5-ON need to be alert toward its future progression to PNS. The latter two patients had clinical manifestations that had some overlap with seronegative NMOSD, but the detection of the CRMP5 antibody changed the following intervention strategy. Therefore, patients with some features of NMOSD, MOGAD or MS with an atypical optic neuropathy combined with retinitis or vitritis should be evaluated for CRMP5 antibodies.

No guidelines exist for the treatment of CRMP5-ON. Local or systemic applications of glucocorticoids, plasma exchange, IVIG, and immunosuppressants have been reported. Most of them are combined with surgery and chemoradiotherapy to intervene the primary tumor. In a cohort, 58% of patients died within 5 years, with a median follow-up of 9.5 months (8). In our cohort, all patients survived at the last time of follow-up (ranged 1.4 to 28.7 months). Three patients had surgery during the initial hospitalization, and were stable during the follow-up. Only two patients who received IVMP improved after the treatment, one patient had worsening non-ocular neurologic symptoms during the steroid taper. After the papillary thyroid carcinoma removed by surgery, the patient stabled. Treatment like IVIG and IVMP may be helpful for visual recovery in a short term, but long-term therapy requires intervention for primary cancer. Therefore, early cancer screening is crucial for prognosis.

This study had a few limitations. A single cohort, small sample size, and short follow-up period hindered the evaluation of the prognosis and relapse rate over the course of the disease. Another limitation was the retrospective design, leading to a certain bias in the data collection. However, this study provided more detailed clinical characteristics of the largest group of patients with CRMP5-ON in China to date. Prospective studies with a larger sample size and longer follow-ups are warranted to confirm our findings.

In conclusion, atypical optic neuropathy seronegative for AQP4 and MOG antibodies, combined with vitreitis, retinitis, or uveitis, should prompt serological testing for CRMP5 antibody. CRMP5-ON can present with MRI optic nerve or perineural optic nerve enhancement, especially in the optic nerve sheath, early investigation might increase sensitivity of MRI. CRMP5-ON also has many neurologic manifestations closely related to PNS. Cancer screening and intervention are crucial to prognosis. Further investigation is needed for a comprehensive understanding of this disease.

## Data availability statement

The original contributions presented in the study are included in the article/supplementary material, further inquiries can be directed to the corresponding author.

## Ethics statement

The studies involving human participants were reviewed and approved by the ethics committee of Beijing Tongren Hospital. Written informed consent for participation was not required for this study in accordance with the national legislation and the institutional requirements.

## Author contributions

RY, YM, and LL contributed to conception and design of the study. RY, YM, HZ, QL, HJ, JP, QY, and SC organized the database. RY performed the statistical analysis and wrote the first draft of the manuscript. YG and JW revised sections of the manuscript. All authors contributed to the article and approved the submitted version.

## Funding

Early diagnosis and prognostic factors of MOG antibody positive demyelinating optic neuropathy, Capital Health Development Scientific Research Project (2020-2-2056, from 2020-07-01 to 2023-06-30). Study on the mechanism of PD-1/PD-L1 and SOX1 in paraneoplastic associated anti-GABABR encephalitis, The National Natural Science Foundation of China (82271384, from 2023-01 to 2026-12).

## Conflict of interest

The authors declare that the research was conducted in the absence of any commercial or financial relationships that could be construed as a potential conflict of interest.

## Publisher’s note

All claims expressed in this article are solely those of the authors and do not necessarily represent those of their affiliated organizations, or those of the publisher, the editors and the reviewers. Any product that may be evaluated in this article, or claim that may be made by its manufacturer, is not guaranteed or endorsed by the publisher.
